# Recent Research on Methods to Improve Tumor Hypoxia Environment

**DOI:** 10.1155/2020/5721258

**Published:** 2020-12-02

**Authors:** Xiao-Hua Zhu, Jun-Xi Du, Dan Zhu, Shen-Zhen Ren, Kun Chen, Hai-Liang Zhu

**Affiliations:** ^1^The Joint Research Center of Guangzhou University and Keele University for Gene Interference and Application, School of Life Science, Guangzhou University, Guangzhou 510006, China; ^2^State Key Laboratory of Pharmaceutical Biotechnology, School of Life Sciences, Nanjing University, Nanjing 210023, China; ^3^Key Laboratory of Molecular Biophysics, Hebei Province, Institute of Biophysics, School of Sciences, Hebei University of Technology, Tianjin 300401, China

## Abstract

Cancer is a major disease burden worldwide. In recent years, in addition to surgical resection, radiotherapy and chemotherapy are recognized as the most effective methods for treating solid tumors. These methods have been introduced to treat tumors of different origins and stages clinically. However, due to insufficient blood flow and oxygen (O_2_) supply in solid tumors, hypoxia is caused, leading to decreased sensitivity of tumor cells and poor therapeutic effects. In addition, hypoxia will also lead to resistance to most anticancer drugs, accelerate malignant progress, and increase metastasis. In solid tumors, adequate O_2_ supply and adequate delivery of anticancer drugs are essential to improve radiotherapy and chemotherapy sensitivity. In recent decades, the researches on relieving tumor hypoxia have attracted researchers' extensive attention and achieved good results. However, as far as we know, there is no detailed review of the researches on alleviating tumor hypoxia. Therefore, in this contribution, we hope to give an overview of the researches on methods to improve tumor hypoxia environment and summarize their effect and application in tumor therapy, to provide a methodological reference for the research and development of new antitumor agents.

## 1. Introduction

As a major public health problem worldwide, cancer can threaten human health seriously. In recent years, although the incidence and mortality of cancer have been alleviated, cancer itself has been a research hotspot in the field of biomedicine due to its complicated pathogenesis, poor treatment effect, and high risk of recurrence [1–3]. In addition to surgical resection, radiotherapy and chemotherapy are our main methods of treating cancer. However, due to the influence of the tumor microenvironment (TME), these treatments have not met people's expectations for good tumor treatment effect, high survival rate, small side effects, and good prognosis [4–6]. The most prominent characteristic of the tumor microenvironment is hypoxia [7, 8].

Under normal physiological conditions, the average partial pressure of oxygen (O_2_) is 40 mmHg. If the pressure is less than this level, a hypoxic environment occurs [9]. Hypoxia is a common pathophysiological characteristic of most solid tumors. The origins of hypoxia can be traced in large measure to the abnormal neovascularization, poor blood flow, and increased proliferation activity of tumor cells, which results in an imbalance between O_2_ supply and O_2_ consumption in tumor cells. Furthermore, tumor hypoxia mainly occurs in the central areas of most solid tumors, such as liver cancer, cervical cancer, and multitype glioblastoma [10, 11]. Tumor hypoxia is also one of the dominant causes of tumor resistance to different cancer treatment [12–14]. Before 60 to 65 years ago, Thomlinson and Gray [15] first hypothesized that hypoxia existed in human tumors, and they observed that hypoxia caused resistance to chemotherapy and radiation. Hypoxia in tumors can also reduce radiosensitivity, accelerating malignant tumor progression and increasing tumor metastasis [16]. Besides, abnormal vascular structures in tumors may significantly limit the delivery of anticancer drugs. Overall, adequate O_2_ supply is essential to improve radiotherapy and chemotherapy's sensitivity in solid tumors [17].

In recent years, researchers have tried to solve hypoxia in tumors and made some achievements. Therefore, in this paper, we induced a series of mitigation measures for the tumor hypoxic microenvironment. In addition, the challenges and prospects of reducing tumor hypoxia for the clinical transformation of cancer treatment will be discussed for providing methodological references.

## 2. Therapeutically Delivering Oxygen to Tumor Tissues

### 2.1. Oxygen-Delivering Therapy Based on HBO

O_2_ breathing at a pressure of more than one atmosphere is called hyperbaric O_2_. Specifically, in a high-pressure environment, artificial methods are used to give O_2_ to the body several or even dozens of times under normal pressure. The human body breathes O_2_ into the lungs and then diffuses it into the blood through alveoli and capillaries. Then, the blood circulation transports O_2_ to other various parts to overcome the hypoxic state [18]. HBO (hyperbaric oxygen) is a treatment in which the body breathes pure O_2_ into more than one atmosphere to increase the amount of O_2_ in the plasma, thus overcoming hypoxia [19]. There are usually two ways of transporting O_2_ in the blood, chemical bonding and physical dissolution. O_2_ in the form of physical dissolution in the blood can increase linearly when the partial pressure of O_2_ rises, but it is rarely affected by other factors. HBO is not limited by the amount of Hb (hemoglobin) on account of it increasing the amount of O_2_ in the tissues by increasing the level of physically dissolved O_2_ [20]. Although the controversy about HBO therapy for cancer patients has been ongoing in the past few decades, studies so far failed to prove that HBO could promote tumor cell growth or cancer recurrence [19, 21]. On the contrary, many studies have shown that HBO therapy is an effective treatment method in clinical and experimental models. This strategy of O_2_ delivery can enhance the amount of dissolved O_2_ in plasma, increasing the pO_2_ in cancer tumor tissue, improving the hypoxic microenvironment of the tumor, and making tumor cells recover their sensitivity to chemotherapy and radiotherapy, thereby generally improving the treating effect [19, 22]. In the process of using HBO, the impact on tumors is mainly manifested in the following aspects: First, HBO changes the spatial position of DNA peptide chains by generating oxygen-free radicals, increasing the sensitivity of anticancer drugs and breaking DNA peptide chains, which shows a synergistic effect with anticancer drugs. Second, hyperbaric oxygen can cause many cells in the G_0_ phase to enter the proliferation phase, increasing the sensitivity to chemotherapy drugs. Third, it reduces the activity of tumor metabolizing enzymes. Fourth, it can increase the permeability of tumor cell membranes and the blood-brain barrier [23].

Given this characteristic of HBO, Peng et al. [24] took liver cancer cells as a model to study the effect HBO on the antitumor drug Sorafenib *in vitro*. This study showed that HBO increased the partial O_2_ pressure of tumors and enhanced Sorafenib's therapeutic effect. Lu et al. [25] studied the antitumor drug Nimustine and HBO's synergistic effect in a nude mouse model of human glioma. The experimental results showed that HBO significantly increased tumor cell sensitivity to Nimustine, thereby significantly improving its antitumor effect. In addition, Hartmann et al. [26] used rhabdomyosarcoma R_1_H as a model to study the HBO effect on radiotherapy. The study showed that HBO promoted tumor oxygenation and improved R_1_H tumor cell sensitivity to radiotherapy, thereby enhancing the effects of tumor radiotherapy. Furthermore, studies showed that the O_2_ partial pressure in normal tissues decreased rapidly after HBO treatment, but in the tumor tissues, it could be maintained for a while due to O_2_ consumption and reduced blood flow [27, 28]. This also explained why hyperbaric O_2_ could be used to relieve hypoxia in tumors.

### 2.2. Treatment Based on Oxygen Delivery of Blood Substitutes

Human blood's primary physiological function is to supply O_2_ to tissues and to take away CO_2_ through reversible binding and separation of O_2_ and CO_2_. To overcome the shortcomings of insufficient blood source and short blood storage time, blood substitutes with O_2_ carrying function have become a research hotspot to increase the O_2_ content of tissues. This strategy also provides a new research method for alleviating hypoxia in tumors. At present, the typical blood substitutes mainly include hemoglobin and perfluorocarbon [17, 29].

#### 2.2.1. Hemoglobin-Based Oxygen Carriers (HBOCs)

With the research of Hb's unique reversible O_2_-binding properties and lack of blood type antigens, purified Hb has been sought as a possible universal substitute for red blood cells [30, 31]. As a blood substitute, hemoglobin transports O_2_ by forming stable local chemical coordination bonds between O_2_ molecules and heme iron atoms, thus realizing the delivery of O_2_ through covalent binding [32]. After a long research period, in current clinical trials, hemoglobin-based O_2_ carriers can be chemically or genetically “engineered” by Hbs to produce desirable O_2_ unloading properties and prolong circulating half-life. Current HBOCs as red cell substitutes are listed in Table 1 [33]. In recent years, the researches on HBOCs have been continuously deepened, and new progress has been made. Funaki et al. [34] described the synthesis and O_2_ affinity of transgenic human adult hemoglobin (rHbA) covalently coated with recombinant human serum albumin (rHSAs) as a fully synthetic red blood cell (RBC) substitute for an artificial O_2_ carrier. The wild-type rHbA (wt) expressed by Pichia coli had the same amino acid sequence and three-dimensional structure as the natural HbA. Replacing Leu-b28 with Trp reduced the distal space in the heme pocket, resulting in a cluster with a moderate hypoxic affinity similar to human RBC. Studies showed that replacing Leu-b28 with Trp could produce a P50 value similar to human RBC, thereby effectively reducing O_2_ affinity. Therefore, these kinds of carriers are very suitable for O_2_ transport *in vivo*. Hence, genetic engineering rHbA(X)-rHSA_3_ cluster is expected to be a new O_2_ delivery product, which can be used to alleviate tumor hypoxia. Blood substitutes have a useful O_2_ transport function, which can deliver O_2_ to tumor tissues. It is expected to improve the tumor hypoxia microenvironment, increase tumor cells' sensitivity to tumor drugs, and thus improve the antitumor effect of the drugs.

#### 2.2.2. Perfluorocarbon-Based Oxygen Carriers (PFBOCs)

As a common blood substitute, perfluorocarbon (PFC) is a class of chemical compounds in which fluorine atoms replace hydrogen atoms in hydrocarbons. Perfluorocarbons (PFCs) are colorless, odorless, nontoxic, transparent liquids with stable chemical properties but insoluble in water, so they need to be emulsified into a soluble emulsion. Moreover, the emulsion has a useful function of dissolving nonpolar gas and can be used as a carrier for O_2_ and CO_2_ [29, 32, 35]. PFCs are chemical combinations of O_2_ transport in the blood. Chemical binding is carried out by binding to hemoglobin (Hb) to form oxygenated hemoglobin (HbO_2_), and the amount of chemically bound O_2_ does not increase with increased pressure after hemoglobin saturation [18].

Perfluorooctylbromide (PFOB) is a type of PFC. It has been widely studied for its excellent dispersion, low surface tension and viscosity, and high gas solubility. Li et al. [36] loaded the chemotherapy drugs Erlotinib and PFOB with the liposome complex as the carrier to investigate the effect of PFOB on the antitumor activity of Erlotinib. The results showed that PFOB promoted the recovery of tumor cells' sensitivity to drugs, overcame the hypoxia-induced lung cancer drug resistance, and improved the antitumor effect of Erlotinib.

With the development of antitumor researches, PFC has been used in other new antitumor therapies due to its unique O_2_-carrying function to alleviate hypoxia and improve the antitumor effect. Cheng et al. [37] used liposomes as carriers for the targeted delivery of photosensitizers IR780 and PFC. This case showed high permeability and retention effect to achieve accumulation in the tumor tissue. A large amount of O_2_ was physically dissolved by PFC in this system, providing O_2_ for the photosensitizer photodynamic treatment process and ensuring the deepening of PDT. Among them, PFC delivered O_2_ to the tumor microenvironment to restore the sensitivity of tumor cells to PDT, thus further improving the antitumor effect. Although PFC has a good solubility for O_2_, it only depends on the O_2_ concentration gradient to release O_2_ through diffusion with low release efficiency. In order to improve the O_2_ release effect of PFC, Song et al. [38] used high-intensity ultrasound as a trigger, aiming to improve the O_2_ release efficiency. The researchers modified PFC nanoemulsions with human serum albumin, which was used in combination with PDT and radiotherapy. They triggered the release of O_2_ in the PFC nanoemulsions by high-intensity ultrasound to study the effect of PFC on tumor resistance. The experimental results showed that the rapid release of O_2_ in PFC by high-intensity ultrasound could effectively reverse the drug resistance related to tumor hypoxia, therefore significantly improving PDT and radiotherapy's therapeutic effect.

## 3. Boosting Tumor Blood Flow

In recent years, using nanomaterials' rich physical and chemical properties, several strategies have been proposed to improve hypoxia TME by boosting tumor blood flow. Promoting the tumor's blood flow and changing the tumor perfusion can also be regarded as an excellent treatment to relieve hypoxia. Through researches, there are two main treatment methods for promoting blood flow in tumors: hyaluronidase and rhythm [39].

### 3.1. HAase

Hyaluronic acid (HA) is a crucial component of the extracellular matrix (ECM) [40]. The main function of HA is to provide a hydrated gel-like matrix to support tumor growth [41, 42]. It has been reported that HA is highly expressed in tumors, and its expression level is positively correlated with tumor grade, the possibility of distal metastasis, and overall survival [43, 44]. Hyaluronidase (HAase), an enzyme that breaks down hyaluronic acid at specific sites, has been used for years as an adjuvant to chemotherapy because it enhances drug penetration [45–47]. Recently, the researches on HAase have also made new achievements. The researchers examined the effects of HAase administration on tumor blood vessels, blood perfusion, and oxygenation. They also checked the synergistic effect of HAase nanoparticle-based PDT therapy with tumor therapy. It was found that both the tumor vascular densities and effective vasculature areas were increased after HAase administration, inducing enhanced perfusion inside the tumor and alleviated the hypoxia state. In addition, they also improved the efficacy of PDT *in vivo* by changing the tumor microenvironment inherent in the primary tumor and metastatic lymph nodes, which was conducive to expanding the clinical application of PDT [48].

### 3.2. Metronomic Chemotherapy

**Figure 1:**
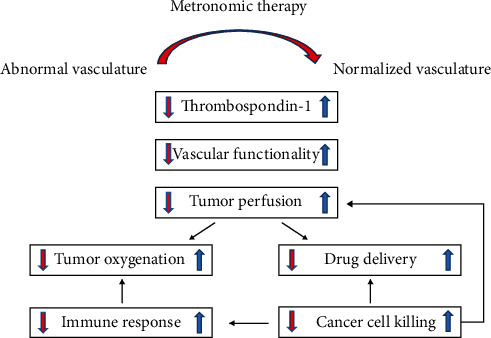
Schematic of the proposed mechanism of action of metronomic therapy.

Metronomic chemotherapy is a treatment in which chemotherapy drugs are frequently administered at doses below the maximum tolerated dose (MTD) and with minimal disruption without drugs [49–51]. It has an antiangiogenic effect on the tumor vascular system, which may be mediated by increasing the level of endogenous angiogenesis inhibitor thrombospondin-1 (TSP-1) [52]. In 2017, Mpekris et al. [53] demonstrated that rhythmic chemotherapy could normalize tumor vascular function and improve tumor perfusion through mathematical models. Subsequently, improved perfusion could enhance drug delivery to solid tumors and reduce hypoxia. These effects could also enhance the immune response and improve the ability to destroy cancer cells, including some more resistant cancer stem cell-like cells. The mechanism of metronomic therapy is shown in Figure 1. Commonly, the metronomic therapy first increases TSP-1 levels and induces tumor vascular normalization, which increases tumor perfusion and oxygenation. Second, improved perfusion increases the proliferation of cancer cells. Besides, improving perfusion and oxygenation can improve the chemotherapy delivery and enhance immune effects, respectively. Finally, more cancer cells are killed, which decompresses tumor blood vessels, further increases tumor perfusion, and forms a positive feedback loop.

Metronomic therapy is also a treatment for relieving tumor hypoxia, but it has not been extensively studied. However, the previous research results will also provide a reference for subsequent research and provide theoretical support for better resolving tumor hypoxia in the future.

## 4. In Situ Oxygen Production

In recent years, in situ O_2_ production has attracted wide attention of researchers and has been applied to the field of PDT. Next, we will summarize various in situ O_2_ production methods in recent years and their applications in PDT or other fields.

### 4.1. Catalyzing Hydrogen Peroxide to Produce Oxygen Based on Nanometer Enzyme

Due to the abnormal blood vessels in tumor tissues and the fact that most tumor cells are located far away from tumor blood vessels, the O_2_ supply is insufficient. Therefore, the two methods mentioned above to improve tumor hypoxia microenvironment have certain limitations. In recent years, researchers have been developing a new type of nanodelivery system which can catalyze the decomposition of endogenous H_2_O_2_ in tumors to produce O_2_, thereby increasing the O_2_ content in tumor tissues and improving the tumor's hypoxia microenvironment.

Reactive oxygen species (ROS) are the single-electron reduction product of O_2_ in the body. They are a general term for chemical active oxygen metabolites and their derivatives. These kinds of metabolites are produced by the reduction of molecular O_2_ by an electron [54, 55]. In normal cells, the oxidative and antioxidant systems are maintained in a relatively balanced state. An increase in prooxidation levels or a decrease in antioxidant capacity will lead to the rise of ROS content in the body. Due to abnormal metabolism, tumor cells have higher ROS levels than normal cells and often in a state of oxidative stress, which results in a higher sensitivity to ROS than normal cells. ROS plays a decisive role in tumor growth and has two sides to tumor growth. Initially, ROS aid tumor growth via DNA damage and uncontrolled proliferation of a genomically unstable and highly aggressive cell line. However, excessive ROS is toxic to tumor cells, leading to cell damage such as lipid peroxidation, DNA adduct formation, protein oxidation, and enzyme inactivation, and ultimately leads to cell death. This can be explained by the “threshold effect” whereby the level of ROS in tumors is usually at sublethal doses. Beyond this level, antioxidants will not be able to perform their normal functions, which will cause cytotoxicity, leading to irreversible damage and apoptosis. Although the accumulation of ROS in tumor cells can lead to the occurrence and continuous development of cancer, they can also become significant targets for tumor treatment [56–59]. H_2_O_2_ is located near the superoxide anion and hydroxyl radicals and is a crucial component of ROS. The increase of intracellular H_2_O_2_ concentration is a unique biochemical characteristic of tumor cells, which also lays the foundation for catalyzing H_2_O_2_ to alleviate tumor hypoxia and to increase the level of ROS in tumors [54, 55, 60].

The decomposition of O_2_ can be improved by the H_2_O_2_-produced O_2_ content of tumor tissues. On the one hand, it can enhance the hypoxic environment of tumors and is expected to overcome the hypoxic-induced cell resistance, thereby improving the effectiveness of drug treatment. On the other hand, the generated O_2_ can also provide adequate O_2_ supply for the treatment of PDT and enhance the antitumor effect of PDT.

#### 4.1.1. Manganese Dioxide Nanoparticles

On the one hand, the amount of H_2_O_2_ in tumors is much higher than that in normal cells. On the other hand, manganese dioxide nanoparticles (MnO_2_ NPs) are highly reactive to O_2_ produced by H_2_O_2_ and will decompose under acidic pH [55–58, 61–64]. Taking advantage of this catalytic property of MnO_2_, in the past four years, people have developed a large number of new nanoscale materials to relieve the hypoxia of tumors and have used them for antitumor therapy. In 2016, Chen et al. [65] designed intelligent multifunctional pH/H_2_O_2_-responsive HSA-coated MnO_2_ nanoparticles through albumin-based biomineralization of Mn^2+^, named HSA-MnO_2_-Ce6&Pt (HMCP) nanoparticles. In this system, HSA was premodified with chlorine e6 (Ce6) as a photosensitizer or (cis-Pt(IV)SA) as a prodrug of cis-platinum and was then used as a template and coating molecule to induce the formation of MnO_2_ nanoclusters through biomineralization under alkaline conditions. The HMCP nanoparticles took advantage of the pH/H_2_O_2_ reaction characteristics of MnO_2_. On the one hand, they reacted with H_2_O_2_ in the tumor to produce O_2_ in situ, overcoming the PDT resistance related to tumor hypoxia. On the other hand, HMCP nanoparticles would gradually degrade into small therapeutic albumin-drug complexes under TEM conditions, which could significantly enhance intratumoral permeability and further improve the therapeutic effect of combined photodynamic chemotherapy. In the same year, Yi et al. [66] applied MnO_2_ to antitumor radiotherapy. They developed gold@manganese dioxide (Au@MnO_2_) core-shell nanoparticles with a polyethylene glycol (PEG) coating as a novel radiosensitizing agent. In this Au@ MnO_2_ nanostructure, the MnO_2_ envelope triggered the breakdown of endogenous H_2_O_2_ in the tumor microenvironment to produce O_2_, overcoming hypoxic-related radiotherapy resistance. Both *in vivo* and *in vitro* experiments have demonstrated that Au@MnO_2_-PEG nanoparticles could significantly improve the antitumor effect during radiotherapy and were an effective radiosensitizer. Animal experiments indicated the low toxicity of MnO_2_. Therefore, their work suggested a novel radiosensitizer with the potential to enhance the treatment of hypoxic tumors. In addition, the research laboratory also studied MnO_2_ in combination with chemotherapy-photodynamic therapy and cancer radiotherapy in the next two years. In 2017, they [67] developed a biodegradable hollow manganese dioxide (H-MnO_2_) nanointelligence platform and obtained the H-MnO_2_-PEG/C&D which could dissociate at a lower pH value of TME. The modified nanoplatform could induce the decomposition of tumor endogenous H_2_O_2_ while releasing the loaded therapeutic molecules. It was used not only for specific imaging of TME and drug releasing on-demand but also for adjusting the O_2_ concentration in TME to enhance tumor treatment effect, which was conducive to the integrated effect of antitumor immune response. In 2018, they [68] designed a core-shell tantalum oxide @ manganese dioxide (TaO_x_@MnO_2_) nanostructure, an effective radiosensitizer for enhancing radiotherapy. Among these nanostructures, the TaO_x_ nucleus acts as a radiotherapy sensitizer which could effectively concentrate X-ray radiation energy into the tumor, while the MnO_2_ envelope could trigger the decomposition of endogenous H_2_O_2_ in the tumor microenvironment to produce O_2_, thereby overcoming the tumor's hypoxia. Their research has broad application prospects in tumor radiotherapy.

Now, with more and more extensive researches, based on the characteristics of MnO_2_-catalyzing H_2_O_2_, MnO_2_ has been mainly applied to the following aspects. First, MnO_2_ was combined with Au, PEG, MoS_2_, WS_2_, etc. to enhance the effect of radiotherapy [66, 69]. Second, the most extensive application of MnO_2_, a nanoenzyme, has been applied to the treatment of PDT and achieved good antitumor effects [70–77]. Finally, some studies have also loaded it with DOX into nanoparticles for combined therapy [78]. Among these treatments, MnO_2_ played a vital role in alleviating tumor hypoxia.

To improve the efficiency of MnO_2_-catalyzing H_2_O_2_ to produce O_2_, Pan et al. [79] recently designed and manufactured a cancer cell membrane-camouflage nanoreactor to continuously produce O_2_ for use in combination with photodynamic-starvation therapy. The nanoreactor achieved continuous O_2_ production through a subordinate reaction: the MnO_2_ scaffold reacted with endogenous H_2_O_2_ to produce O_2_. Glucose could be oxidized to H_2_O_2_ by GO_X_, and the generated H_2_O_2_ could provide enough O_2_ for subsequent reaction of MnO_2_ and H_2_O_2_. The reaction process is shown below.

(1) $$\begin{array}{c} {\text{MnO}}_{2}+{\text{H}}_{2}{\text{O}}_{2}+2{\text{H}}^{+}\to {\text{Mn}}^{2+}+2{\text{H}}_{2}\text{O}+{\text{O}}_{2}↑ \end{array}$$

(2) $$\begin{array}{c} \text{Glucose}+{\text{O}}_{2}\underset{\to }{\text{ G}{\text{O}}_{\text{X}}}\text{ Gluconic acid}+{\text{H}}_{2}{\text{O}}_{2} \end{array}$$

**Figure 2:**
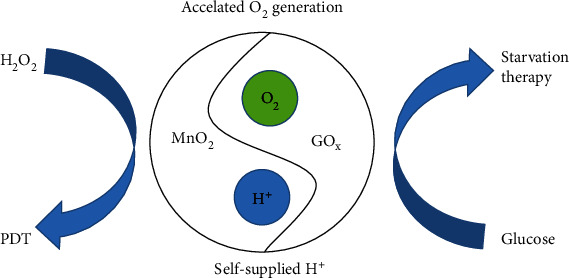
The mechanism diagram of biomimetic hybrid nanozyme (named rMGB).

Yang et al. [80] also tried to mutually promote the natural enzyme (bigger) and glucose oxidase enzyme nanometer MnO_2_ and to develop a biomimetic hybrid nanozyme (rMGB), which could maximize MnO_2_ with bigger enzyme activity (Figure 2). Their research results raised the ability and efficiency of MnO_2_ to catalyze H_2_O_2_, to better alleviate tumor hypoxia, and to lay a good foundation for subsequent further research on MnO_2_ in reducing tumor hypoxia and antitumor therapy.

#### 4.1.2. Calcium Peroxide Nanoparticles

In addition to MnO_2_ NPs, calcium peroxide nanoparticles (CaO_2_ NPs) are another type of substance that reduces hypoxia. Unlike MnO_2_ which directly catalyzes the production of O_2_ from H_2_O_2_ in tumors, CaO_2_ reacts with water in tumors to produce H_2_O_2_ and then indirectly generates O_2_ through the decomposition of H_2_O_2_. CaO_2_ is a more effective source of H_2_O_2_ than liquid H_2_O_2_ [81, 82]. It dissolves to form H_2_O_2_ and calcium hydroxide (Ca(OH)_2_), releasing the maximum H_2_O_2_ [83]. However, only a particular nanosized calcium peroxide can improve the surface-to-volume ratio, increasing the reaction speed [84].

People have been trying to find a simple and effective method to synthesize this kind of high efficient CaO_2_ NPs, but no suitable way has been found. In 2011, Khodaveisi et al. [85] found that by improving the surface areas in the synthesis of nanosized calcium peroxide, the reaction speed could be accelerated, and the problem of slow oxidation reaction of calcium peroxide was solved. They developed a simple surface modification technique to avoid irreversible agglomeration of calcium peroxide nanoparticles. The technology was based on the hydrolyzation-precipitation process, using CaCl_2_ as the precursor and polyethylene glycol 200 (PEG200) as the surface modifier. It was characterized by XRD, TEM, and so on. The results showed that this method could synthesize new nanoscale reagents, and the TEM image measurement showed that the size of calcium peroxide nanoparticles was about 15-25 nanometers, which improved the rate of in situ chemical oxidation. Their results on the synthesis of CaO_2_ NPs laid an essential foundation for the future use of CaO_2_ NPs to alleviate tumor hypoxia and antitumors.

Based on the above-mentioned synthetic methods, the reaction rate of CaO_2_ NPs has been greatly increased. People have tried to apply them in the field of antitumors, and many new important research results have been obtained. It is well known that in the absence of adequate O_2_, cancer cells grown in hypoxic solid tumors are resistant to antitumor drugs (such as doxorubicin, DOX) due to reduced ROS production in the cells. In 2016, Huang et al. [86] used HBO therapy to improve the O_2_ content of hypoxic tumor tissue, thereby increasing the sensitivity of the tumor cells to DOX. Still, the combination of HBO and DOX also enhanced ROS-mediated drugs' effect on normal tissue cytotoxicity. Given this situation, they proposed an idea that local O_2_ treatment with implanted O_2_-generating depot could enhance the cytotoxicity of DOX to malignant tissues in a highly site-specific manner without increasing the level of systemic O_2_. When implanted near the tumor, the O_2_-producing reservoir reacted with the mesenchymal medium to produce O_2_ in situ, which effectively reduced the hypoxic zone in the tumor tissue and increased the local O_2_ supply. This procedure would lead to a significant increase in the toxicity of the oxidoreductase in tumor cells and eventually reduce the resistance of solid oxidoreductase caused by hypoxia in malignant tumors. Importantly, this increased cytotoxicity was limited to the tumor site, which would greatly reduce the side effects of cancer treatment. They designed the O_2_-generating depot by dropping an alginate solution containing CaO_2_ and catalase into a calcium chloride (CaCl_2_) bath to form Ca^2+^ crosslinked microcapsules, which were then filtered and air-dried. Upon implantation close to the tumor, the CaO_2_ that is encapsulated in the alginate pellets reacts with the water that infiltrates the pellets from the interstitial tissues to produce calcium hydroxide [Ca(OH)_2_] and hydrogen peroxide (H_2_O_2_). Some of the generated H_2_O_2_ would decompose naturally, and the other part was catalyzed by the catalase added in the alginate pellets to decompose H_2_O_2_ into O_2_ molecules rapidly, while the Ca^2+^-crosslinked polymeric alginate matrix prolonged O_2_ production by restricting water molecules' penetration into the pellets, thereby increasing the therapeutic effect of DOX and CaO_2_/hydrolysis reactions of catalase activity. Their research ideas provided a reference for the follow-up local relief of tumor hypoxia.

**Figure 3:**
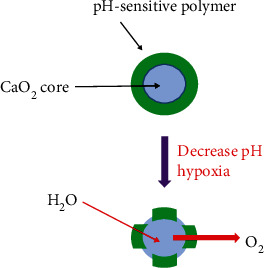
A schematic diagram of the reaction of nanoparticles in different pH environments is designed, in which green represents the polymer coating and blue represents the nanoparticles.

Inspired by the results of the previous studies, in the past two years, more and more researchers have focused on CaO_2_ NPs and applied them to alleviate hypoxia in tumor treatments, especially in PDT. In 2017, Sheng et al. [87] prepared a formulation of CaO_2_ NPs coated with a sensitive polymer so that the production of molecular O_2_ was controlled by pH. The polymer coating was designed to protect particles from being broken down during circulation but could be activated under low pH conditions inside the tumor (Figure 3).

The designed polymer produced only a small amount of O_2_ at a pH of 7.4 but could significantly increase the production capacity of O_2_ at a pH of 6.2. Polymer-coated CaO_2_ NPs were also observed to increase tumor pO_2_ levels in mice significantly. PDT-mediated efficacy also improved statistically in the same tumor mice after microparticle therapy 20 min before PDT (*p* < 0.001). These results indicated that polymer-coated CaO_2_ NP formulation could provide great potential for in situ O_2_ production and could improve the therapeutic effect by relying on the presence of O_2_ to induce cytotoxic effects. In the same year, Liu et al. [88] also applied CaO_2_ NPs to the treatment of PDT by designing liposome-based nanoparticles (designated LipoMB/CaO_2_) with the properties of O_2_-independent dual-stage optical drive PDT. First, after a short time of irradiation, ^1^O_2_ activated by methylene blue (MB) photosensitizer could cause lipid peroxidation to destroy liposomes, so that the contact area between CaO_2_ and H_2_O increased, thus accelerating the generation of O_2_. The accelerated production of O_2_ could further modulate the hypoxic tumor microenvironment, thereby increasing the production of ^1^O_2_ in MB under another long time irradiation. The *in vivo* and *in vitro* experiments also confirmed that LipoMB/CaO_2_ had great advantages in reducing hypoxia, inhibiting tumor growth and antitumor metastasis, and had fewer side effects. This double-path light-operated self-sustaining LipoMB/CaO_2_ nanometer platform was a successful PDT attempt to treat hypoxic tumors, which laid the foundation for future researches. In the second year, Ji et al. [89], inspired by the previous two results and MnO_2_, combined CaO_2_, MnO_2_, and MB for the first time to alleviate hypoxia in tumors. A novel multifunctional nanosystem CaO_2_/MnO_2_@ polydopamine- (PDA-) methylene blue (MB) nanosystem (CMP-MB) was designed. First, CaO_2_ NPs were coated with MnO_2_ NPs, and then PDA was coated on the surface of CaO_2_/MnO_2_ NPs, which can be adsorbed by hydrophobic action or p-p accumulation of adsorbent photosensitizer MB. In this nanosystem, CaO_2_/MnO_2_ had the ability to produce cytotoxic effects on its own depending on the presence of O_2_, which largely solved the problem of tumor hypoxia. In addition, the fluorescence of MB could be inhibited by MnO_2_ and activated in the simulated tumor microenvironment. Therefore, CMP-MB nanofilms were expected to be used for on-off control of cell imaging. *In vitro* cell experiments proved that CMP-MB nanofilms could achieve tumor microenvironment response imaging and effectively inhibit tumor cells' growth under the light. This result suggested great potential for PDT applications and on-off imaging of tumor cells.

It is precisely because of the previous research foundation that more and more research results have been obtained. In the past two years, there have been more and more reports on the application of CaO_2_ in antitumor trials. In addition to PDT treatment, CaO_2_ NPs have been also used in the combination of chemotherapy/chemotherapy dynamic therapy, tumor immunochemotherapy, and other fields [90–93].

Especially in 2019, Wang et al. [93] prepared a self-oxygenating/biodegradable inorganic nanozyme with a core-shell structure to alleviate tumor hypoxia during cancer immunochemotherapy. By integrating biocompatible CaO_2_ as an oxygen storage element, this strategy delivered O_2_ or H_2_O_2_ more efficiently than the nanocarriers designed earlier, thus providing significant O_2_ cogeneration and long-term reduction of hypoxia in tumor tissues. They believed that this was a simple, reliable, and effective strategy to improve tumor hypoxia using the decomposition and biocompatibility of inorganic nanometer enzyme reactors. It not only provided an innovative way to reduce tumor hypoxia but also inspired other cancer treatments with O_2_ or provided references for the treatment of diseases caused by the lack of O_2_.

#### 4.1.3. Ferroferric Oxide Nanoparticles

Magnetic nanoparticles have long been considered biologically and chemically inert. Most research focused on the high-efficiency separation capabilities of magnetic nanoparticles, such as modified enzymes, catalysts, and surface antibodies to achieve biological or chemical catalysis [94, 95]. In 2007, Gao et al. [96] discovered for the first time that ferroferric oxide nanoparticles (Fe_3_O_4_ NPs) had a catalytic activity similar to that of peroxide-mimicking enzymes, and its catalytic performance was consistent with natural horseradish peroxidase (HRP), which could catalyze H_2_O_2_ to produce highly active hydroxyl radicals (^·^OH) and O_2_ [97, 98]. The procedure is mainly manifested in the following aspects: First, in the presence of H_2_O_2_, Fe_3_O_4_ NPs can interact with HRP substrates 3,3′,5,5′-tetramethylbenzidine (TMB), diazine benzene (DAB), o-phenylenediamine (OPD), etc., and blue, brown, and orange reactions occur, respectively. The resulting reaction products are the same as those of HRP. Second, the catalytic activity of Fe_3_O_4_ NPs is related to pH, temperature, catalyst concentration, and H_2_O_2_ concentration. In addition, the catalytic mechanism of Fe_3_O_4_ NPs is consistent with HRP. The double inverse curve showed that its catalytic mechanism was similar to the ping-pong mechanism of HRP's enzyme-catalyzed reaction, that is, Fe_3_O_4_ NPs reacted with the first substrate first and then reacted with the second substrate after releasing the first product. The results showed that, compared with HRP, Fe_3_O_4_ NPs could withstand high concentrations of H_2_O_2_ and could still maintain higher catalytic activity in a larger pH and temperature range.

One of the most extensive properties of Fe_3_O_4_ NPs is that they convert excess H_2_O_2_ into highly toxic ROS-hydroxyl radicals (^·^OH) and exhibit oxide-like activity under acidic conditions to achieve antitumor effects [96, 99]. However, there are few studies on using Fe_3_O_4_ NPs to catalyze H_2_O_2_ to O_2_ for biological applications. Based on the facts that Fe_3_O_4_ NPs can catalyze the decomposition of H_2_O_2_ into nontoxic H_2_O and O_2_ under neutral pH conditions and exhibit similar activity to hydrogen peroxide, researchers have also begun to try to use Fe_3_O_4_ NPs to reduce hypoxia in tumors and conduct antitumor treatments.

In 2018, Zhang's research team [100] investigated the ability of Fe_3_O_4_ NPs to catalyze H_2_O_2_ to produce O_2_ for antitumor use. The remarkable feature of Fe_3_O_4_ NPs was to use the simultaneous production of ^·^OH as a therapeutic element and use the in situ production of O_2_ to regulate the tumor hypoxic microenvironment to overcome the limitations of photodynamic therapy. To this end, they designed an ROS activation platform which used the high reactivity of peroxide-like Fe_3_O_4_ to endogenous H_2_O_2_ while producing ^·^OH as a therapeutic element to provide O_2_ for O_2_-dependent PDT. Chitosan-coated nano-Fe_3_O_4_ nanoparticles were prepared and modified with CuS and porphyrins (FCCP NPs). On the one hand, multimode imaging could be achieved; on the other hand, O_2_ could be generated to relieve hypoxia and to enhance the therapeutic effect of tumors. Modified nanoparticles (FCCP NPs) showed strong endogenous peroxidase-simulated activity. It was easy to track the tumor aggregation characteristics of FCCP NPs after intravenous injection through multimode *in vivo* imaging including photoacoustic imaging (PAI) and magnetic resonance imaging (MRI). Both *in vitro* and *in vivo* research results showed that FCCP NPs could kill cancer cells very effectively through the combination of therapy and photothermal therapy. Their research work proved that nanomaterials could be used in PDT to promote the generation of ROS and O_2_, which was expected to overcome the shortcomings of current cancer treatment methods.

In addition to Fe_3_O_4_ NPs, other ferromagnetic nanoparticles, such as *γ*-Fe_2_O_3_, FeS, CoFe_2_O_4_, and Mn_x_Fe_3-x_O_4_, all contain Fe^2+^/Fe^3+^ catalytic activity centers and have peroxidase-simulated enzyme catalytic activity [101–104]. However, there is no other research on ferromagnetic nanoparticles in the treatment of tumor hypoxia. With the increase of research numbers, the use of ferromagnetic nanoparticles such as Fe_3_O_4_ NPs to relieve tumor hypoxia may become a good choice for antitumor applications in the future.

#### 4.1.4. Cerium Oxide Nanoparticles

At present, except for the ferromagnetic nanoparticles above, CeOx nanoparticles (CeOx NPs) due to advantages such as low toxicity, catalytic, adjustable absorption spectra and trivalent oxidation state, and tetravalent oxidation state between the advantages of convenient transformation have also aroused people's attention [105–108]. It is well known that CeOx NPs exhibited different enzyme activities according to the ratio of Ce^3+^ to Ce^4+^. However, in the +4 state, the higher the cerium content is, the more obvious the effect of catalase will be, accompanying the more sustainable and effective the inhibition effect on tumor hypoxia [109–111]. Nevertheless, in our investigation of the literature, we found that people paid more attention to the +3 state of cerium ions to alleviate inflammation and mimic enzyme activity [112, 113], while CeOx NP catalase activity was rarely applied to the treatment of tumor hypoxia. Fortunately, so far, some researchers have studied the application of catalase-like activity CeOx in antitumor therapy and achieved some scientific results.

Fan et al. [114] developed a smart photosensitizer cerium oxide nanoprobe for the first time and applied the catalase-like activity of CeOx to biotherapy, which became a promising example of high-performance photodynamic therapy. In this study, they designed and assembled an intelligent stimulus-response nanoprobe (CeOx-EGPLGVRGK-PPa) on the basis of the CeOx nanooctahedron modified with the photosensitizer pyropheophorbide-a(PPa)-labeled peptide (EGPLGVRGK-PPa) and further used them to improve tumor selectivity and reduce tumor hypoxia. When the tumor biomarker MMP-2 disconnects the peptide ligand (EGPLGVRGK), the smart nanoprobe can switch from a “silent state” before reaching the cancer cell to an “active state” in the cell, emit fluorescence, and produce ^1^O_2_. In this design, CeOx was used to decompose endogenous H_2_O_2_ to produce O_2_, which reduced tumor hypoxia. Through the routine application of CeOx, researchers have innovatively demonstrated how smart nanoprobes could relieve tumor hypoxia to achieve highly selective and effective personalized treatment. The reaction equations are represented as follows:

(3) $$\begin{array}{c} {\text{H}}_{2}{\text{O}}_{2}+2\text{C}{\text{e}}^{4+}\to 2{\text{H}}^{+}+{\text{O}}_{2}+2\text{C}{\text{e}}^{3+}\\ {\text{H}}_{2}{\text{O}}_{2}+2\text{C}{\text{e}}^{3+}+2{\text{H}}^{+}\to +2{\text{H}}_{2}\text{O}+2\text{C}{\text{e}}^{4+} \end{array}$$

Their research results provided a useful reference for the future use of CeOx to alleviate tumor hypoxia. Subsequently, Jia et al. [115] also published their findings on using CeOx in antitumor studies. By wrapping a mesoporous cerium oxide (mCeOX) on upconversion nanoparticles (UCNPs, NaGdF4: Yb, Tm-NaGdF4), the team prepared a hollow-structure biophotocatalyst. The catalyst used near-infrared (NIR) lasers to decompose H_2_O_2_ in the tumor microenvironment and produced O_2_ to improve PDT effect. Long-wavelength near-infrared lasers have low phototoxicity to the inert shell of the biological tissue and core. The structure of UCNPs could effectively convert near-infrared photons into ultraviolet (UV) light, thereby exciting the photocatalytic effect of CeOx on PDT. Also, the internal space of UCNPs@mCeOx was able to store the chemotherapy drug adriamycin (DOX), thus enhancing the synergistic effect of PDT and chemotherapy. Animal experiments have shown that nanomaterials could reach the tumor through enhanced permeability and retention (EPR) effects when injected into tumor-bearing mice through the tail vein. It was of great significance that the huge hollow structure could effectively load the chemotherapy drug DOX and realize the coordinated treatment of PDT and chemotherapy. This strategy had an excellent anticancer effect and broad application prospects. Although CeOx NPs have good endogenous H_2_O_2_ catalytic activity, single use could not meet the requirements of modern tumor treatment. Therefore, people have been working hard to find more effective ways to relieve tumor hypoxia and antitumor treatment. Fan et al. [116] combined CeOx and Fe_2_O_3_ NPs based on previous studies and successfully developed a sea urchin-like hollow CeOx/Fe_2_O_3_-C&D as an intelligent TME-responsive nanoprobe for combined therapy. Because the CeOx/Fe_2_O_3_ carrier had a sea urchin-like cavity structure, the chemotherapeutic DOX and the photosensitizer chlorine e6 (Ce6) were coloaded to form a CeOx/Fe_2_O_3_-C&D nanoprobe. In the tumor's TME environment, when the CeOx/Fe_2_O_3_ nanocell began to break, DOX was released rapidly at the tumor site. CeOx/Fe_2_O_3_ exhibited catalase activity, decomposed endogenous H_2_O_2_ into H_2_O and O_2_, and continuously injected O_2_ to overcome in situ hypoxia. Their research demonstrated an innovative strategy that combined multiple approaches to alleviate hypoxia in tumors, thereby increasing cellular uptake capacity, regulating hypoxia in tumors, and achieving highly selective and specific combination therapy. Simultaneously, it provided a template for the preparation of a stimulation-responsive nanoprobe and provided a scientific reference for the specific diagnosis and treatment of hypoxic tumors in clinical applications.

Through investigations and reviews of peroxide nanoparticles to relieve tumor hypoxia, it is not difficult to find that MnO_2_ NPs and CaO_2_ NPs are the current research hotspots. There are few studies on Fe_3_O_4_ NPs and CeOx NPs, but in recent years, some researchers have begun to carry out related studies on the relief of tumor hypoxia and have achieved some research results. We believe that in the near future, as research continues to deepen, researchers will find better ways to alleviate tumor hypoxia and even discover new oxide nanoparticles which can be used as a kind of nanoenzyme for antitumor therapy.

#### 4.1.5. Platinum Nanoparticles

With the further development of nanoenzymes, it has been discovered that metal nanoparticles also have enzyme simulation properties and have been widely used in biomolecular detection, antibacterial, ROS elimination, and environmental monitoring. In particular, platinum nanoparticles (Pt NPs), as a well-known catalyst for catalyzing a variety of chemical reactions, have been observed to have enzymatic mimic activity [117, 118].

Previous researches on Pt NPs mainly focused on catalyzing a variety of chemical reactions. In 2015, Zhang et al. [119] first provided a common and simple method for synthesizing mesoporous-MOFs through encapsulation and etching processes. By simply adjusting the type and packaging conditions of the NPs, a good crystal structure was maintained, and its size, shape, and spatial distribution could be controlled through mesopores. In particular, a functionalized mesoporous-MOF-Pt hybrid material was obtained, which had high catalytic activity and good selectivity due to the protection of the microporous framework during the catalytic hydrogenation process. Their method could design and synthesize mesoporous structures with adjustable mesopore sizes and different shapes and functions, which further expanded the application prospects of mesoporous structures and laid the foundation for the application of Pt NPs in the field of antitumors. Xiao et al. [120] studied the relationship between the position of Pt NPs relative to MOFs and the photocatalytic efficiency. The results showed that the encapsulated Pt NPs had higher efficiency due to the short electron transfer path and the avoidance of unnecessary volume charge recombination. Their research not only showed a deeper understanding of the electron transfer mechanism of metal nanocomposites but also provided a unique perspective for the development of efficient MOF-based photocatalysts and even other porous materials. Their research on the material itself led to the biological application of Pt NPs in alleviating tumor hypoxia. Later, the research team became interested in the potential of nanomaterials as biomimetic enzymes. They studied the potential of biocompatible Pt NPs as antioxidant nanozymes and carefully evaluated the cytotoxicity, cytocompatibility, and cellular uptake ability of Pt NPs. It proved that Pt NPs had strong and extensive antioxidant properties, similar to or better than natural enzymes, and had a strong ability to adapt to changes in environmental conditions. These results also laid a vital foundation for the further application of Pt NPs in tumor cells.

In 2018, Zhang et al. [121] successfully developed a multifunctional PDT-enhanced nanometer platform, which modified Pt NPs to photosensitizers integrated with MOFs. The modified Pt NPs on MOFs had high activity and stability similar to hydrogen peroxide, which could catalyze H_2_O_2_ in tumor cells to produce O_2_ and to promote the formation of cytotoxic ^1^O_2_ at hypoxic tumor sites, thereby causing more serious damage to cancer cells and improving the treatment efficiency of PDT. Their research identified the potential of nanoenzymes and MOFs in combination as effective drugs for the treatment of cancer and laid the foundation for their application in modern oncology. Later, a simple and effective strategy for precise control of the crystal size of MOFs was developed by preloading the ligand precursor with a small number of metal nodes to form amorphous clusters, separating nucleation and growth processes and regulating them separately [122]. When the total metal node precursors were added, these clusters acted as crystalline seeds, grew rapidly, and formed MOF crystals. By changing the *R* value, the number and density of seed crystals could be adjusted to precisely control the size of the MOF crystal. This study provided new opportunities for understanding the basic principles of crystallization, studying MOFs, and determining the high activity of multifunctional MOFs. What was more, this also provided a reference for the application of Pt NPs to MOF materials to obtain higher catalytic activity to alleviate tumor hypoxia. In antitumor researches, to better relieve tumor hypoxia and obtain better treatment effect, Liu et al. [123] combined Pt NPs with a porous gold nanometer shell, prepared platinum-nanozyme encapsulated NH_2_-MOFs, and combined it with the porous gold nanometer shell and photosensitizer Ce6 to make it a photosensitizer nanoparticle with continuous O_2_ production capacity (Pt@UIO-66-NH_2_@Aushell-Ce6). Therefore, it was used together with PDT and PTT to treat tumors and offered a good synergistic tumor treatment strategy.

In the past two years, more and more researchers have applied Pt NPs to alleviate tumor hypoxia and tried to design a variety of nanomaterials containing Pt NPs for antitumor research. Including in 2018, researchers [124] designed a multifunctional Pt NP-based core-shell nanometer platform as a nanofactory to enhance tumor therapy. The treatment platform consists of a dopamine nucleus, a platinum-nanoparticle interlayer, and a zirconium porphyrin (PCN) shell. This is a core-to-shell hybrid nanostructure that provides necessary products at different times and spaces. Pt NPs can catalyze the overexpressed H_2_O_2_ in tumors to produce O_2_ and then convert O_2_ into ROS through the PCN shell under light irradiation, thus enhancing the PDT effect. In addition to improving PDT, continuous O_2_ production can also reduce tumor hypoxia and inhibit tumor metastasis. This kind of stable and efficient nanometer platform provides new ideas for more effective tumor treatment and better prognosis.

In 2019, Pt NPs have been applied to sonodynamic therapy (SDT), and good therapeutic effects have also been achieved. Since the efficacy of SDT requires the assistance of O_2_, they [125] designed and synthesized a new platinum-copper alloy composed of a hollow semiconductor copper and precious metal platinum. The inner cavity could be filled with sonosensitizer molecules (tetramer (4-aminophenyl) porphyrin, TAPP) to achieve SDT. In addition, on the one hand, platinum deposition improved the photothermal properties; on the other hand, it also catalyzed endogenous decomposition of H_2_O_2_ to produce O_2_, which could overcome tumor hypoxia and effectively promote the apoptosis of cancer cells. Notably, under the irradiation of an 808 nm laser, Cu could accelerate the catalytic activity of Pt, improve the O_2_ level, and further promote the efficacy of SDT. In addition, after coating the temperature-sensitive copolymer p (OEGMA-co-MEMA), the activity of the nanoenzyme and the drug release rate could be intelligently controlled by temperature. In this study, under the intervention of Pt NPs, the synergistic effect of PTT and SDT catalytic enhancement was realized, and the tumor can be completely eradicated without obvious recurrence. Their simple and versatile nanofoil platform provided a new paradigm for anticancer and a wide range of biomedical applications. Yang et al. [126] prepared a multifunctional nanometer preparation consisting of cisplatin-loaded, dopamine-coated, and GE11 peptide-bound superparamagnetic iron oxide nanoparticles (GE11-PDA-Pt-USPIOs) for tumor hypoxia and MRI/PAI-guided tumor radiotherapy. Among them, the free iron ions released by USPIOs responded to the tumor's acidic microenvironment, leading to the decomposition of endogenous H_2_O_2_ in tumor, effectively alleviating the hypoxia state of the tumor, and enhancing the effect of radiotherapy.

With the deepening of the researches, people used Pt NPs to catalyze H_2_O_2_ to generate O_2_ to alleviate tumor hypoxia, continuously tried to design various effective tumor treatment platforms, and continuously optimized and upgraded the designed structure to achieve better antitumor effects. For example, Liu's team [127] proposed a dual-enzyme engineering porphyrin metal-organic framework-driven in situ catalytic cascade coprocessing strategy, especially Pt NPs sandwiched between PCNs to simulate the catalase. Then, it was embedded in ultrafine Au NPs that mimicked glucose oxidase and further interacted with folic acid (P@Pt@p-Au-FA). Pt NPs could convert H_2_O_2_ in tumors into O_2_, effectively alleviate tumor hypoxia, significantly enhance antitumor effects, and prevent tumor recurrence and metastasis. Compared with other peroxide nanoparticles, Pt NPs have stronger antioxidant properties and better catalytic properties than natural enzymes and have a strong ability to adapt to changes in environmental conditions. Their research results provided a method for further researches on nanoscale enzymes and a means for designing catalytic cascade models with practical application value. Similarly, Liang et al. [128] reported a multifunctional nanometer drug delivery system PDA-Pt-CD-@RuFc, which was modified by CD and loaded with Ru(II) complex through host-guest interaction. The nanometer platform could accumulate in tumor tissues and had the ability to image in multiple modes such as photothermal, PA, and CT, which showed great potential for PDT-PTT-combined therapy. This nanometer platform mainly alleviated tumor hypoxia from the following aspects: First, Pt NPs could catalyze H_2_O_2_ to produce O_2_. Second, the vasodilation caused by photothermal heating could maintain O_2_ supplementation. Finally, PDT applied by RuFc could also occur through O_2_-independent Fenton reaction. Their design idea provided a good basis and reference for relieving tumor hypoxia later.

### 4.2. The Production of Oxygen from Hydrogen Peroxide Is Catalyzed by Natural Enzymes

As mentioned above, due to the advantages of nanoenzymes themselves, artificial nanoenzymes are currently widely used to relieve hypoxia in tumors. However, in addition to nanoenzymes, the natural enzymes also play an essential role in alleviating the hypoxia of tumors.

#### 4.2.1. Catalase

As we all know, catalase (CAT) is an enzyme that catalyzes the decomposition of H_2_O_2_ into O_2_ and H_2_O and is a natural enzyme found in cell peroxides. Catalase has been widely used in the field of antitumor because of its natural O_2_-producing properties.

In 2016, Cheng et al. [129] developed an O_2_-like self-contained cell-like biomimetic nanometer platform (CAT-PS-ZIF@Mem) that contained molecules embedded in CAT protein. When intracellular H_2_O_2_ penetrates into the skeleton, it was catalyzed by CAT to produce O_2_ at the hypoxic tumor site, thereby promoting the production of toxic ^1^O_2_. The combination of CAT to the nanometer platform could alleviate the hypoxia of tumors and show a highly specific and effective PDT for hypoxic tumor cells, thus significantly reducing the side effects on normal tissues. The combination of CAT and nanoframework has established a tremendous impact on the field of antitumors. Subsequently, studies [130] indicated that CAT could maintain its biological function under a wider range of conditions by embedding it into MOF microcrystals by de novo synthesis. This was because the enzyme molecules were confined in the mesopores of the MOFs, which reduced the structural fluidity of the enzyme molecules. Their results proved that the combination of CAT and MOFs could play a better role in reducing tumor hypoxia. In 2017, Cai et al. [131] established a core-shell intelligent nanocomposite UCNPs/MB@ZIF-8@CAT as an efficient nanocomposite, which suggested biological imaging and efficient PDT functions. In this design, the high porosity of ZIF-8 provided an effective platform for adsorbing O_2_ molecules catalyzed by endogenous H_2_O_2_, thereby promoting the generation of ^1^O_2_ and improving the efficiency of PDT. This was the first example of an intelligent nanocomposite that leveraged UCNPs and MOFs to design an efficient PDT for hypoxic tumors and opened up new ways for the use of MOF materials in effective cancer therapy. It provided a reference for a more efficient combination of CAT and MOFs.

Afterwards, more and more researchers tried to use the combination of catalase and MOF to design nanoscale drugs to alleviate the hypoxia of tumors and to carry out tumor treatment, thus achieving good antitumor effect [98, 132–135].

Although only using catalase to relieve hypoxia can achieve good results, it is far from meeting the needs of clinical cancer. Therefore, a combination of measures to reduce tumor hypoxia has emerged at the right moment. In 2017, a team of researchers [136] developed a unique biocompatible nanodelivery system called HSA-Ce6-CAT-PTX, which contained the chemotherapeutic drugs paclitaxel (PTX) and CAT. On the one hand, PTX could improve the ability of tumor perfusion and help reduce tumor hypoxia. On the other hand, CAT in the nanoparticles could cause the decomposition of endogenous TME H_2_O_2_ and generate O_2_ in situ; thus, it could alleviate the tumor's hypoxia and improve the efficacy of the combination of photodynamic therapy and chemotherapy. Their work proposed a simple drug-induced self-assembly strategy to produce enzyme-loaded therapeutic albumin nanoparticles for cancer's coordinated combination therapy. In the process of improving radioimmunotherapy, catalase also achieved good therapeutic effect in the treatment of hypoxia. In 2018, Song et al. [137] developed an innovative strategy to alleviate hypoxia by introducing exogenous H_2_O_2_ into the tumor and then triggering the decomposition of H_2_O_2_ by catalase. In this strategy, H_2_O_2_ and CAT were separately loaded into invisible liposomes. First, the CAT@liposome vein was injected, then the H_2_O_2_@liposome was injected 4 h later. CAT@liposome could degrade the sustained release of H_2_O_2_ and can achieve the lasting effect of enhancing tumor oxygenation. With the addition of H_2_O_2_, their approach would be more effective than the in situ tumor oxygenation strategy in decomposing only a limited amount of endogenous H_2_O_2_ from the tumor. In addition, compared with the above-mentioned O_2_ carriers (such as PFC and hemoglobin nanoparticles), H_2_O_2_ itself was more efficient as an oxygen precursor because of its higher solubility. Therefore, the combination therapy of CAT@liposome+H_2_O_2_@liposome could significantly enhance the therapeutic effect of cancer radiotherapy. By using mature liposome carriers, they deliver CAT and exogenous H_2_O_2_ to the tumor in turn, thereby promoting tumor oxidation and providing a good idea and platform for alleviating tumor hypoxia. This also showed great clinical transformation potential in cancer radioimmunotherapy.

Biomolecules encased in a metal-organic framework can protect biological functions in harsh environments. Although this method (called biosimulated mineralization) is successful, considering the MOF coating's chemical properties is still limited. In the process of antitumor therapy, in order to better protect and play catalase activity, a team of researchers [138] recently proved that enzymes encapsulated in hydrophilic MAF-7 or ZIF-90 could retain enzyme activity while those encapsulated in hydrophobic ZIF-8 could not maintain enzyme activity. There was almost no protective effect on urease in high temperature, denaturing or proteolytic agents, and organic solvents. Their results indicated the importance of ZIF and ZIF/biointerfaces in promoting and protecting the encapsulated enzyme's biological function. Recent reports indicated that optimizing the hydrophobic/hydrophilic interaction between enzymes and polymers was critical for effective encapsulation and stabilization of biomolecules. Due to its modular synthesis, porosity, and chemical and structural diversity, MOFs represented a new type of materials that needed to be further explored in the field of biomolecular protection. Researches on this new material provided the possibility for peroxidase and other O_2_-producing materials to play a better role in future.

In short, these results indicated that the combined use of catalase and MOFs could give full play to the catalytic capacity of H_2_O_2_ to produce O_2_ in tumors and help to reduce hypoxia in tumors to the maximum extent.

## 5. Decreasing the Oxygen Consumption of Cancer Cells

In recent years, hypoxia has been considered as a negative factor leading to drug resistance in tumors, and several studies have shown that hypoxia can promote tumor survival [139–141]. Hypoxia-inducible factor-1 (HIF1) is a well-recognized transcription factor characterized by hypoxia, which can regulate tumor growth, metastasis, angiogenesis, etc. In order to overcome the lack of O_2_ during chemotherapy, attempts have been made to increase the supply of O_2_ to the tumor site [141–144]. In the method of reducing tumor hypoxia, in addition to increasing the O_2_ supply for tumors, the researchers also adopted a reverse method to increase oxygenation by reducing the O_2_ consumption of cancer cells, which also provided another scientific research idea for alleviating hypoxia of tumors.

### 5.1. Metformin

At present, metformin (Met) has been found to be effective in reducing the O_2_ consumption of tumors. Met is an oral hypoglycemic agent widely used in the treatment of type II diabetes. It has been proven to be an effective respiratory inhibitor that inhibits cellular respiration by directly inhibiting the activity of the mitochondrial electron transport chain complex I [145–150]. In 2013, Zannella et al. [148] reported that intraperitoneal injection of Met could reduce O_2_ consumption in tumors and effectively improve tumor oxygenation response to radiotherapy. The results laid the foundation for further application of Met to antitumor treatments. It was only later that Met was used to the treatment of PDT. In 2016, a team of researchers [151] used Met for the first time to reduce O_2_ consumption in nanomedicine-mediated PDT treatment. They developed a liposome-based drug carrier system Ce6 and diabetes drug Met, in which hydrophilic Met and a modified hydrophobic Ce6 (HCe6) were encapsulated in the inner and outer membranes of liposomes, respectively. In this carrier system, PEGylated liposomes increased the targeted delivery of Met to tumors and continue to release, thereby reducing O_2_ consumption in tumor cells and significantly enhancing the efficacy of PDT. In addition, animal studies showed that mice treated with Met were significantly less hypoxic than untreated mice. Using these drugs to regulate the poor hypoxic tumor microenvironments by reducing the O_2_ consumption of the tumor was a much simpler alternative than existing methods, such as in situ generation of O_2_ in the tumor or the use of O_2_ carriers to deliver O_2_. This new strategy could be combined with other nanotechnology approaches for cancer oxygenation to improve the effectiveness of cancer treatment using O_2_ in the cell killing process. Uehara et al. [152] also found in their research on the treatment of osteosarcoma that the addition of Met therapy reduced the basal respiration and O_2_ consumption (OCR)/extracellular acidification rate (ECAR) ratio of CD11b^+^ cells in tumors, which would enable Met to play a greater role in the field of antitumors.

Based on the previous research results, recent studies on metformin's effectiveness in reducing tumor O_2_ consumption have become more and more extensive, and good research results have been achieved. In 2019, Li et al. [153] achieved corresponding research results in reducing tumor O_2_ consumption by Met. They prepared a cationic liposome for codelivery of DOX and Met, which synergistically acted on MCF7/ADR in multidrug resistant breast cancer. Among them, Met as a mitochondrial inhibitor could reduce the O_2_ consumption of tumors, thus improving the tumor's hypoxic state. DOX-Met lipid enhanced tumor targeting, promoted tumor reoxygenation, and improved treatment efficiency *in vivo*. Their research is aimed at improving the efficacy of multidrug resistance cancer treatment by improving the tumor's hypoxic microenvironment and also provided a feasible strategy for antitumor treatment. In the field of PDT treatment, the previous research strategy is to produce as much O_2_ as possible during PDT treatment to alleviate tumor hypoxia. Now, researchers [154] have applied Met to the treatment of PDT and overcome hypoxia-induced cancer treatment by reducing the consumption of O_2_. They designed a traceable nanoplatform (DOX/Met/BSA-HA-CDs) in which carbon dots (CDs) were used not only as a PDT reagent but also as traceable imaging *in vivo*. The results of animal experiments also showed that combined with PDT and chemotherapy, the tumors in mice injected with DOX/Met/BSA-HA-CD nanoparticles were significantly reduced. Compared with the previous research results, the traceable Nanocatalyst produced by the team is a greater innovation. It could not only improve the efficacy of the combination PDT and chemotherapy by reducing the O_2_ consumption in the tumor hypoxic microenvironment but also raise a good clinical application prospect as a traceable imaging method.

At present, the treatment of tumors is mainly through photodynamic therapy, and alleviating hypoxia of tumors has always been the focus of scientific researchers. On the basis of previous studies, it is of great significance to innovate and combine multiple methods to find better ways to relieve tumor hypoxia. Recently, Jiang et al. [155] developed a multifunctional nanocluster bomb (UCGM NPs) consisting of upconversion NPs, CeOx, graphite-C_3_N_4_ (g-C_3_N_4_) NPs, and Met. In this design, on the one hand, the catalytic effect of CeOx was used to oxidize H_2_O_2_ to O_2_, thereby alleviating hypoxia. On the other hand, the ability of Met to act on mitochondria to inhibit tumor cell respiration could be used to further increase O_2_ levels. At the same time, the central UCNP had a significant photothermal capacity, which could activate g-C_3_N_4_ NPs to generate ROS for cancer treatment at 808 nm. In general, the design of the multifunctional nanoplatform had great potential in imaging guided joint PDT/PTT. At the same time, these UCGM NPs also showed excellent performances in upconversion luminescence, magnetic resonance imaging and computer tomography. These advantages made them a potential image-guided drug delivery system. It was wise for them to apply CeOx and metformin together in scientific research and innovation, and it was worthy of learning from both positive and negative approaches to alleviate tumor hypoxia. This also provided a useful reference for future scientific research in related fields.

In the process of relieving hypoxic tumors, in addition to the efficacy of the substance itself, the carrier used is also very important. Recently, Mai et al. [156] developed a platelet membrane (PM) as a nanomaterial to coencapsulate Met and IR780 (PM-IR780-Met NPs). In this design, due to the active adhesion of PM to tumor cells, a more significant accumulation of IR780 and Met was produced in the tumor, which also led to a longer cycle life of the nanocarriers. The introduction of Met inhibited mitochondrial respiration, reduced the O_2_ consumption of the tumor, and significantly reversed the hypoxia of the tumor, leading to the initiation of O_2_-promoted PDT, the increase of immunogenic cell death (ICD), and the activation of immunogenic pathways. Meanwhile, the involvement of Met in PM-IR780-Met NPs also reversed the immune suppression pathway regulated by bone marrow-derived suppressor cells (MDSC). Finally, a large number of T cells were activated and migrate to tumor tissues, which not only provided a promising treatment method for removing the primary tumor but also opened up new ways for effective ablation of tumor metastasis.

In a word, reducing tumor O_2_ consumption is a new idea to alleviate tumor hypoxia. Although there are not many researches in this area, many researchers have conducted research on this and have achieved good results in the field of antitumors. It is believed that with the deepening of research, this method may become a good method in the field of antitumors in the future, with good development prospects and scientific research value.

## 6. Conclusion and Outlook

Cancer is caused by various factors that disrupt the balance of cell survival, proliferation, and differentiation. However, hypoxia has always been a critical factor influencing the development of drug resistance in cancer treatment and chemotherapy, which leads to the vital role of tumor hypoxia in oncology attracting more and more attention. How to better alleviate the hypoxia of tumors and achieve better antitumor effects has always been the topic of most concern. Therefore, we reviewed various approaches to reduce tumor hypoxia, which could be summarized as therapies that delivered O_2_ to tumor tissues, promoted tumor blood flow, produced O_2_ in situ, and reduced the O_2_ consumption of cancer cells. Among these methods, in situ O_2_ production is currently the most widely used method, which can produce good effects, and most of the substances that O_2_ produces are combined with PDT to enhance antitumor effects. However, at the same time, the production efficiency of O_2_ is still not high, and the persistence is not long enough. Meanwhile, reducing the O_2_ consumption of the tumor is another way to relieve the hypoxia of the tumor, which is also a new research idea with good application prospects. In conclusion, the purpose of this review is to provide references for future research. With the development of scientific research, there is no doubt that finding a better way to relieve tumor hypoxia is of great significance for future antitumor researches. Nevertheless, in the face of the complexity of the tumor microenvironment and the heterogeneity caused by individual differences, it is still a long way to seek a better method to alleviate tumor hypoxia and apply it to the clinical treatment of tumors.

## Figures and Tables

**Table 1:** HBOCs in development as red blood cell substitutes.

Product	Hb source	Technology	Developer	Status
DCL-Hb (HemAssist)	Human red cells	*α*-*α* crosslinked Hb	Baxter Healthcare (Deerfield, IL, U.S.A.)	Phase III (trauma) (suspended)
RHb1.1/1.2 (Optro)	*E. coli*	Recombinant human *α*-*α* fused Hb	Baxter Healthcare (Somatogen <1998)	Phase II (discontinued)
RHb2.0	*E. coli*	Recombinant Hb	Baxter Healthcare	Preclinical
HBOC-201 (Hemopure)	Bovine red blood cells	Glutaraldehyde polymerization	BioPure (Cambridge, MA, U.S.A.)	BLA filed (elective surgery) Approved for clinical use in S. Africa
Human POE-Hb (PHP)	Human red cells	PEG conjugation	Curacyte (Apex) (Munich, Germany)	Phase III (septic shock)
Hb-raffimer (HemoLink)	Human red cells	Oligomerization with o-raffinose	Hemosol (Toronto, Canada)	Phase III (cardiac surgery) (suspended)
Pyridoxal polyHb (PolyHeme)	Human red cells	PLP-Hb polymerized Hb with glutaraldehyde	Northfield Laboratories (Evanston, IL, U.S.A.)	Phase III (trauma) Filed BLA
Hemospan	Human red cells	Conjugated with maleiimide PEG	Sangart (San Diego, CA, U.S.A.)	Phase II (elective surgery)
HemoZyme	Human red cells	Polynitroxylated Hb	SynZyme (Irvine, CA, U.S.A.)	Preclinical
PolyHb-SOD-CAT	Bovine red cells	Hb modified with SOD and catalase	McGill University (Montreal, Canada)	Preclinical
PEG-Hb	Bovine red cells	PEG-conjugated Hb	Enzon (Piscataway, NJ, U.S.A.)	Phase Ia (discontinued)
OxyVita	Human/bovine red cells	Stabilized Hb with sebacoyl diaspirin	IPBL Pharm. (Goshen, NJ)	Preclinical
HemoTech	Bovine red cells	Modified Hb with o-ATP, o-adenosine, and glutathione	HemoBioTech (Amarillo, TX, U.S.A.)	Preclinical

## Data Availability

The data used to support the findings of this study are included within the article.
